# Vitamin D signaling in thymic development and longevity

**DOI:** 10.3389/fnut.2026.1961627

**Published:** 2026-09-15

**Authors:** Patricio Artusa, John H. White

**Affiliations:** 1 Department of Physiology, McGill University, Montreal, QC, Canada; 2 Department of Medicine, McGill University, Montreal, QC, Canada

**Keywords:** autoimmune regulator (AIRE), central tolerance, thymic aging, thymic development, vitamin D

## Abstract

Vitamin D has pleiotropic physiological roles including immune system regulation. Notably, there are links between vitamin D deficiency and risk of autoimmunity. Hormonal vitamin D binds to the vitamin D receptor (VDR) to exert its genomic effects. In immune cells, these include regulation of activation, inflammatory responses, and cytokine production. However, vitamin D supplementation is not efficacious in treatment of existing autoimmune disease. Conversely, vitamin D intake during childhood is associated with reduced risk of developing autoimmune type 1 diabetes. This supports a prophylactic role for vitamin D during this early developmental period wherein T cells undergo maturation in the thymus; importantly, highly self-reactive thymocytes undergo apoptosis due to interactions with specialized antigen presenting cells. Central to this process is the transcription factor autoimmune regulator (AIRE), which induces ectopic expression tissue restricted antigens in medullary thymic epithelial cells. Impaired AIRE function results in multiorgan autoimmunity in mice and humans. Vitamin D signaling regulates Aire expression and function in the mouse and, importantly, supports long-term thymic health by delaying aging-associated thymic involution. In humans, thymic aging is correlated with reduced naïve T cell output, impaired response to infection and cancer, and increased risk of autoimmunity. Collectively, existing data suggest that vitamin D sufficiency throughout the lifespan may be important for generating a self-tolerant T cell repertoire through its effects on AIRE and during aging by delaying aging-associated thymic involution. However, little is known about the links between thymic longevity and vitamin D status in humans, which represents a substantial knowledge gap.

## Vitamin D and the immune system

Vitamin D can be obtained from dietary sources or by cutaneous conversion of 7-dehydrocholesterol (pre-vitamin D) in skin in the presence of adequate solar ultraviolet B irradiation (1). However, western diets tend to be vitamin D deficient and cutaneous synthesis only occurs efficiently when the sun is above 45 degrees (1). This, coupled with sun avoidance and conservative dress in some populations, leads to seasonal variations in circulating vitamin D metabolite levels and widespread vitamin D deficiency in the absence of adequate supplementation (2–4). Vitamin D was discovered for its role in the prevention and treatment of nutritional rickets in children, which arises from inadequate intestinal calcium absorption, leading to defects in bone growth (5). However, numerous studies have identified several extra-skeletal effects of vitamin D, most notably in the innate and adaptive arms of the immune system (5). Associations between vitamin D and immunity date from the 1800’s, when sun exposure was found to be beneficial for treatment of tuberculosis. Since then, vitamin D deficiency has been linked by various randomized controlled trials (RCTs) to susceptibility to infections and autoimmunity (5–8).

To become biologically active vitamin D must undergo sequential hydroxylation events (9, 10). 25-hydroxylation of vitamin D occurs in largely the liver and is catalyzed primarily by CYP2R1, although other cytochrome P450 enzymes such as CYP27A1 catalyze 25-hydroxylation. 1α-hydroxylation to form biologically active 1α,25-dihydroxyvitamin D3 (1,25D) is catalyzed by the enzyme CYP27B1. Initially, 1α-hydroxylation was thought to solely occur in the kidney under control of calcium homeostatic inputs. However, we now know that CYP27B1 is expressed in several tissues with functions unrelated to calcium homeostasis (10–12). Notably, 1α-hydroxylation occurs in several cells of the immune system including macrophages, monocytes, dendritic cells, T cells and B cells. Importantly, regulation of CYP27B1 expression in immune cells is independent of calcium homeostatic inputs (13).

## The thymus

We became interested in the links between vitamin D deficiency and development of autoimmunity, in particular type 1 autoimmune diabetes (T1D), where the association is particularly strong (6, 8). A birth cohort study of 10,366 children born in northern Finland was particularly intriguing in this regard. It found that vitamin D supplementation in infancy was associated with a dramatically decreased incidence of T1D (relative risk of regular versus no supplementation, 0.12) (14). Children taking the recommended dose (2,000 IU/day) had a relative risk of 0.22 compared with those receiving lower doses, whereas infants presenting with symptoms of rickets had a 3.0-fold increased relative risk of T1D development. These findings are particularly intriguing because sun exposure is not a significant source of vitamin D in northern Finland. Moreover, they suggested to us that vitamin D signaling in early childhood may be influencing a critical window in thymic development during which T cell repertoires are being established and filtered for highly self-reactive cells.

T cells derive their name from their obligatory passage through the thymus where they undergo a multistep maturation process. Mature T cells initiate immune responses following binding of foreign antigen or mutated self-peptides bound to major histocompatibility molecules (pMHC) expressed on antigen presenting cells (APCs) via specific T cell receptors (TCRs) (15). TCR production is a semi-stochastic process capable of generating up to 10^15^ unique heterodimeric receptors (16). It is driven by the expression and recombination of variable, diversity, and joining TCR gene fragments followed by non-templated nucleotide addition into gene junctions (17). However, due to the stochastic nature of this process the majority of produced TCRs would be unable to appropriately engage pMHC complexes and undergo apoptosis, a process known as death by neglect (Figure 1) (17). A positive selection process occurs in the thymic cortex, wherein cells with TCRs competent to engage self-pMHC expressed on cortical thymic epithelial cells (cTECs) are retained (18). This leads to MHC restriction of surviving cells. Cells with TCRs of insufficient avidity for self-pMHC undergo apoptosis (18). Although TCRs are positively selected for certain self-pMHC combinations expressed on cTECs, they also display cross-reactivity to foreign-pMHC (19). Moreover, the affinity for self and foreign peptides of individual TCRs are proportional (20). From an evolutionary perspective, this is likely to fill metaphorical holes in the repertoire to prevent pathogen escape (21). All positively selected TCRs display limited reactivity to self-pMHC complexes, which becomes essential for naïve T cell survival and homeostatic proliferation after thymic egress (22). Reactivity to pMHC expressed by professional APCs such as dendritic cells (DCs), macrophages, and B cells is also important for regulation of T cell activation and differentiation during an immune response (22).

**FIGURE 1:**
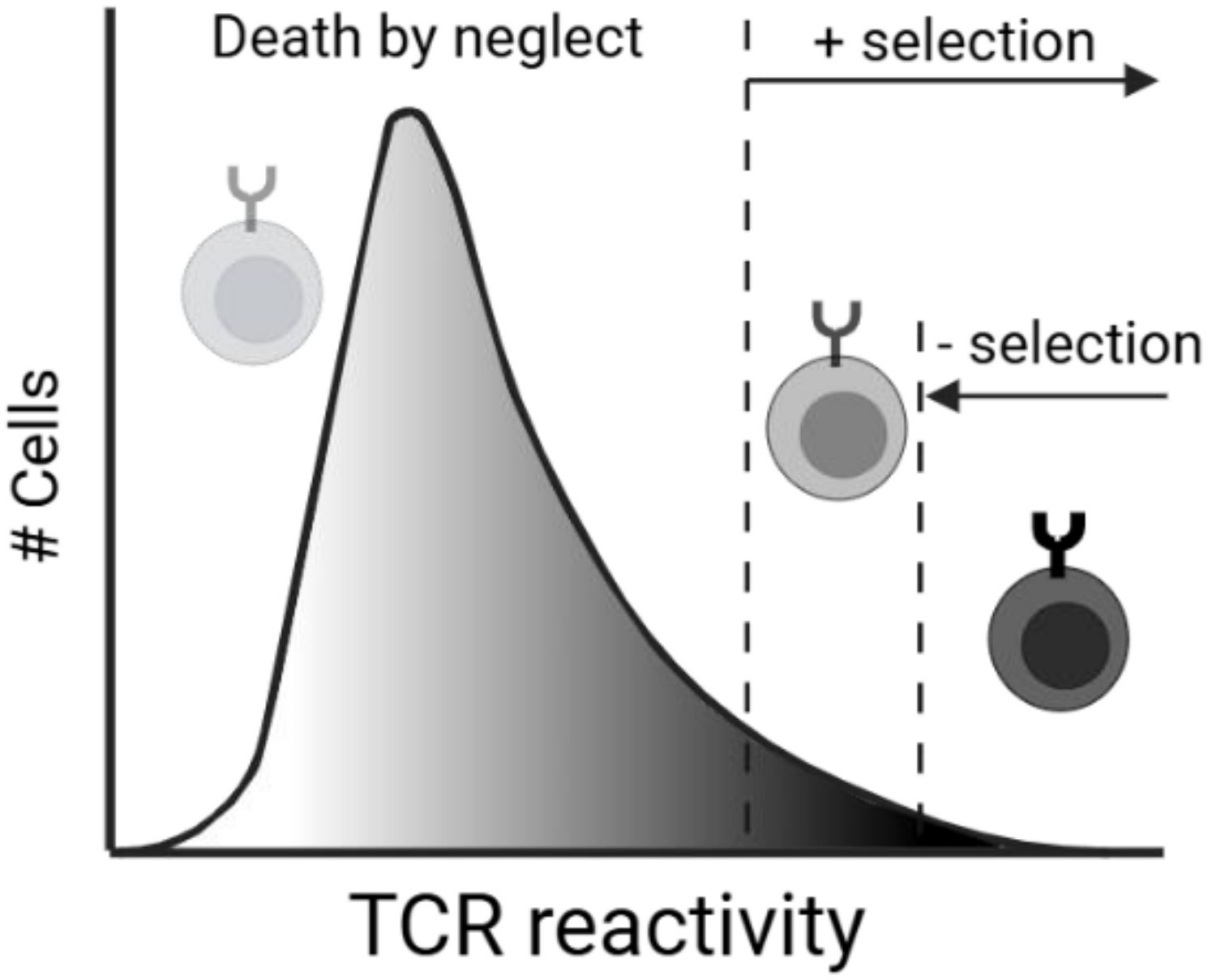
T cell receptors (TCRs) are selected based on their reactivity toward self-pMHC complexes. Cells with sufficient reactivity are positively selected, whereas the majority, those with insufficient reactivity, undergo apoptosis in a process known as death by neglect. Positively selected cells with overly high TCR reactivity are induced to undergo apoptosis in a process known as negative selection.

Autoimmunity arises from the inappropriate activation of T cells arising from higher avidity interactions of TCRs with self-pMHC. Various peripheral mechanisms exist to limit the activity of highly self-reactive T cell clones such as induction of anergy, expression of inhibitory receptors, or TCR downregulation (23). In addition, regulatory T cells (Tregs) suppress autoimmunity via inhibitory cytokine production among other mechanisms (24). However, the primary mechanism to eliminate inappropriate TCR-self-pMHC interactions occurs in the thymus in a process known as central tolerance (23). Positively selected thymocytes from the cortex migrate to the medulla and interact with self-pMHC expressing medullary TECs (mTECs), DCs, or B cells. Cells that undergo strong TCR signaling arising from high avidity self-pMHC interactions are induced to undergo apoptosis (negative selection) or converted into Tregs (23).

An essential component of this process is the presentation by thymic APCs of tissue-restricted antigens (TRAs), which would normally only be found in specific cell types extrathymically (25). This is possible due to the activity of the critical thymic transcription factor, autoimmune regulator (AIRE), which induces the transcription of TRA genes in Aire^+^ mTECs, resulting in the translation and presentation of TRA peptides to thymocytes (Figure 2) (25). The expression and presentation of individual TRAs in the thymus can be directly related to autoimmunity in associated organs. For example, thymic-specific deletion of *Ins2*, which encodes a peptide component of insulin, was shown to result in autoimmune infiltrates into the pancreas in mice (26). More broadly, loss of AIRE function in humans results in autoimmune polyendocrinopathy candidiasis ectodermal dystrophy (APECED) (27). While the clinical presentation varies between individuals, APECED typically presents as multiorgan autoimmunity, notably in endocrine organs, as well as recurrent *Candida* infections due autoantibodies blocking the function of antifungal cytokines such as IL-17 and IL-22 (27). Similarly, genetic deletion of *Aire* in mice results in multiorgan autoimmunity characterized by T cell infiltrates to several organs and autoantibodies (28). Therefore, tolerance to self is first established in the thymus with AIRE at its center and later maintained through peripheral mechanisms.

**FIGURE 2:**
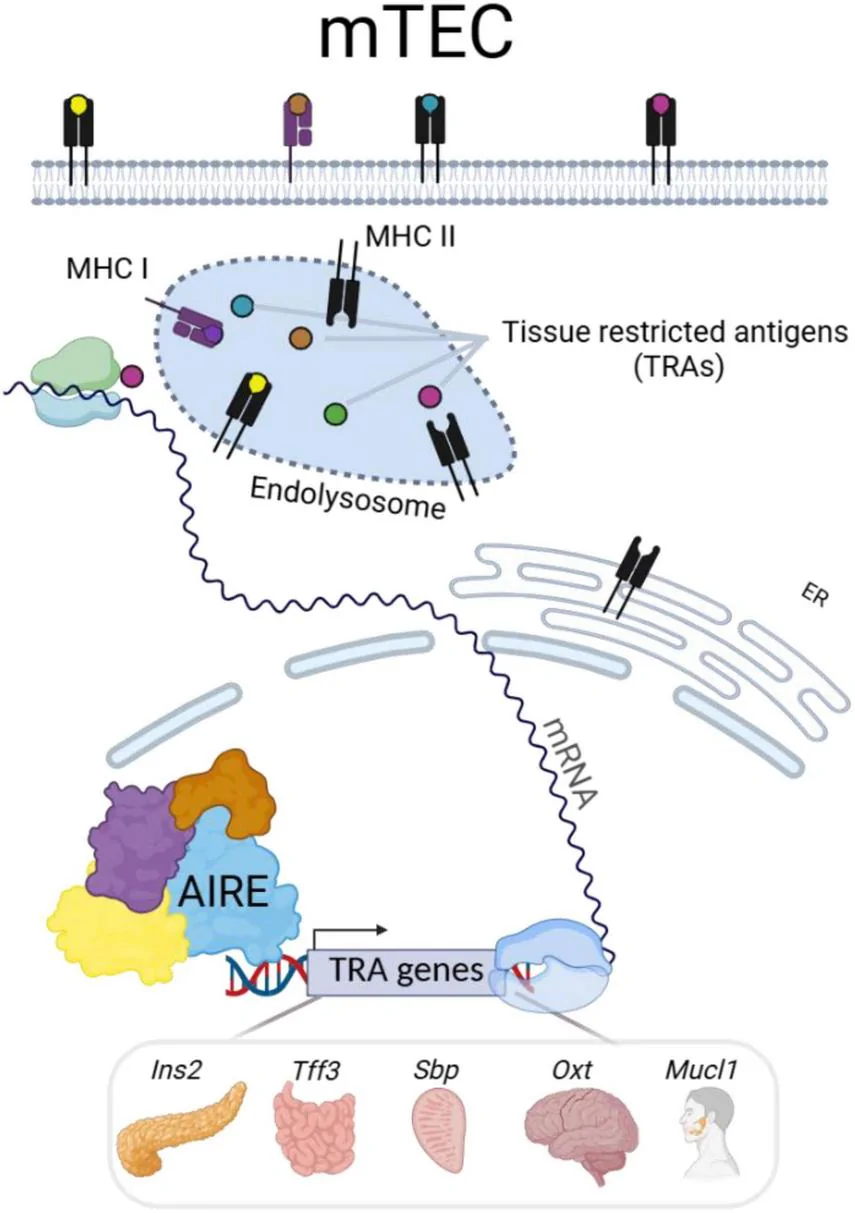
Autoimmune regulator (AIRE) induces the transcription of tissue restricted antigen (TRA) genes in medullary thymic epithelial cells (mTECs). Translated TRAs are loaded onto MHC-I or MHC-II complexes and shuttled to the cell surface for antigen presentation to thymocytes.

Finally, unlike most organs, the thymus begins to rapidly age after adolescence (29). This is characterized by atrophy of the organ, reduced cell counts and diminished naïve T cell output due to a variety of mechanisms including a decline in epithelial cell proliferation, reduced expression of thymic longevity factors, and adipocyte infiltration (Figure 3) (29). This suggests that thymic central tolerance may be particularly important during early life to establish a self-tolerant TCR repertoire. Thus, understanding how environmental and epigenetic cues play into thymic processes may be key to deciphering the development and prevention of autoimmune diseases.

**FIGURE 3:**
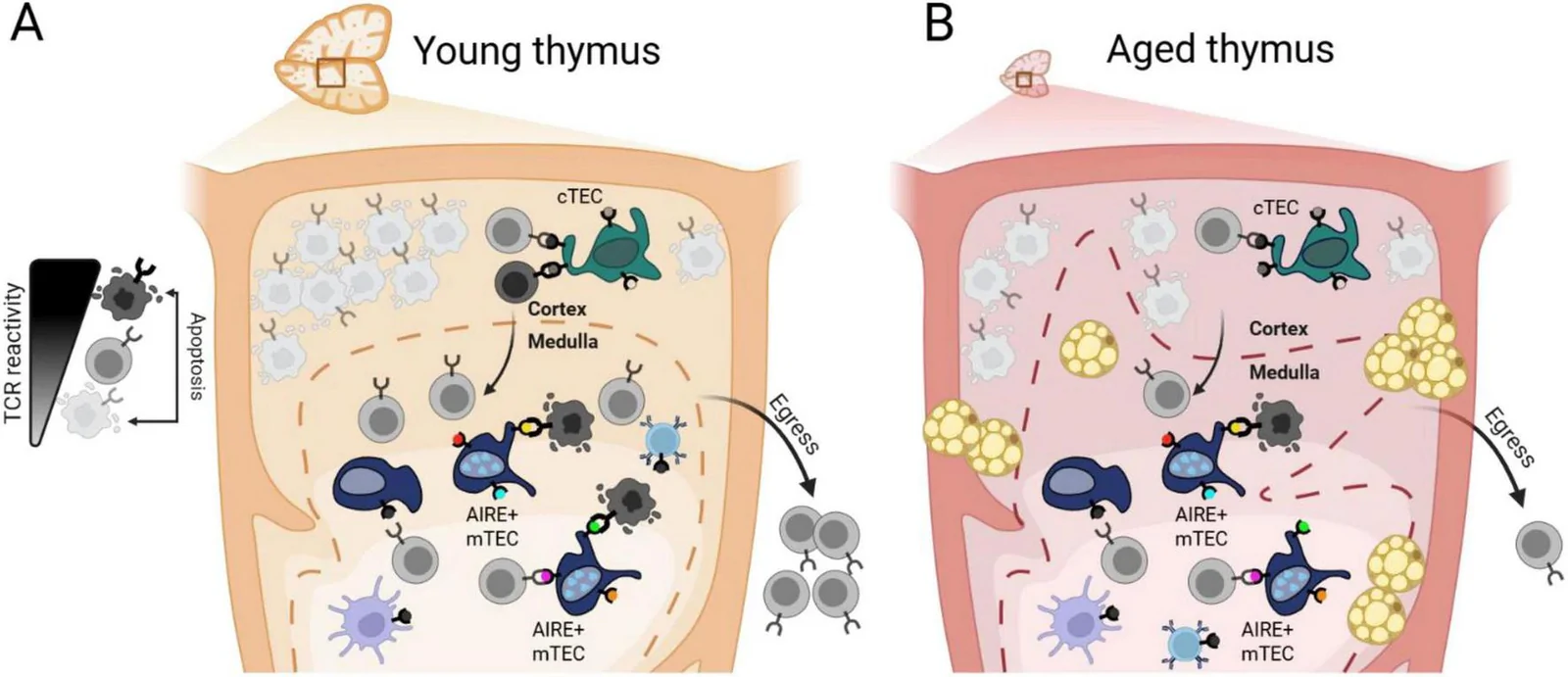
Overview of central tolerance in young and aged thymi. **(A)** Young thymus: thymocytes with low avidity T cell receptors (TCRs) undergo death by neglect in the cortex. Thymocytes receiving positively selecting signals through moderate to high avidity TCR:pMHC interactions with cortical thymic epithelial cells (cTECs) migrate to the medulla. High avidity TCRs are negatively selected by pMHC-expressing AIRE^+/–^ medullary thymic epithelial cells (mTECs), dendritic cells (DCs) and B cells. Cells with moderate avidity TCRs continue maturing and egress from the thymus to the periphery. **(B)** Aged thymus: the thymus undergoes aging-associated involution. This is associated with diminished tissue size, reduced cell counts, adipocyte infiltration into perivascular and later cortical and medullary spaces, blurred cortico-medullary boundaries, impaired negative selection of thymocytes (73, 74), and decreased naïve T cell output.

## Vitamin D signaling in the thymus

While calf thymus was used historically as a source of vitamin D receptor for biochemical studies (30), very little was known about the role of vitamin D in thymic biology. Studies in the past 15 years have revealed a link between vitamin D signaling and the thymic development and function of innate-like thymocyte populations including invariant natural killer T (iNKT) cells and CD8aa+ intraepithelial lymphocytes in mice (IEL) (31, 32). Importantly, conventional thymocytes in the mouse were shown to express low but detectable levels of the Vdr (33–35). However, in vitamin D-deficient mouse models, conventional T cell development appeared minimally altered with relatively unchanged frequencies of mature CD4+ and CD8+ thymocytes or Tregs (32, 36, 37). However, in this regard, it is important to note that the autoimmune phenotype of Aire-deficient mice is not accompanied by substantial changes in thymocyte frequencies (38). Thus, in vitamin D deficiency, there may be functional differences in the reactivities or specificities of the TCR repertoire that predispose to more autoreactive T cells, which bypass negative selection and egress from the thymus.

Indeed, our group found that thymocytes in *Cyp27b1*-deficient mice, which are incapable of producing hormonal vitamin D, had reduced expression of markers associated with negative selection (36). The number of apoptotic (cleaved caspase 3^+^) thymocytes was reduced in the thymic medulla of *Cyp27b1*-deficient mice versus littermate controls. Furthermore, there was reduced expression of Helios in Foxp3^–^ CD4^+^ thymocytes of null mice, a marker upregulated in cells undergoing negative selection. Our group and others have also noted the presence of inflammation in certain organs of aged vitamin D-deficient mice under homeostatic conditions (36). We also noted the presence of autoantibodies in the pancreas and stomach of aged *Cyp27b1*-deficient animals, as well as immune infiltrates in pancreatic islets of Langerhans (36). Adult, but not young, null mice also had impaired glucose clearance as determined by glucose tolerance tests (36), consistent with progressive disease affecting insulin secretion such as T1D Together, these data indicate that there are defects in negative selection in the absence of 1,25D signaling and that this may contribute to the development of T1D.

We explored this idea further by investigating the expression and function of the Vdr in mouse thymic APC populations and found that both the Vdr and Cyp27b1 were expressed in DCs, B cells, and TECs, notably quite highly in Aire^+^ mTECs (34). The presence of Cyp27b1 strongly suggests that the production of 1,25D in the thymus is locally regulated (34). Signaling through the Vdr was confirmed by treating thymic slices, or isolated TECs, with vehicle or 1,25D and measuring the induction of the Vdr-specific target gene *Cyp24a1* (34). Moreover, both the number of *Aire* transcripts and Aire^+^ cells were increased by 1,25D treatment of adult thymic slices for 6 h. Importantly, the expression of a range of TRA genes were also variably induced by 1,25D treatment. Finally, mice deficient for the *Vdr* or *Cyp27b1* had significantly diminished numbers of Aire^+^ cells and reduced mean Aire expression (36). However, the phenotype was attenuated in *Vdr*-deficient mice relative to *Cyp27b1*-deficient animals, potentially due to 1,25D-extrinsic functions of the Vdr. Single cell RNA sequencing data from wildtype versus *Cyp27b1*-deficient TECs confirmed the decrease in Aire^+^ cells and an increase in Aire- mTECs, suggesting skewed mTEC differentiation in the absence of 1,25D signaling. Collectively, these data show that vitamin D contributes to Aire expression and mTEC differentiation into Aire^+^ cells, revealing an important role of vitamin D signaling in shaping central tolerance through the regulation of TRA production (Figure 4). However, it is unclear what the effects of nutritional deficiency, versus genetic depletion of signaling, would be on these processes. This is particularly relevant concerning the potential effects of vitamin D on human thymi (discussed below), because even individuals deficient for vitamin D do not have a complete absence of signaling.

**FIGURE 4:**
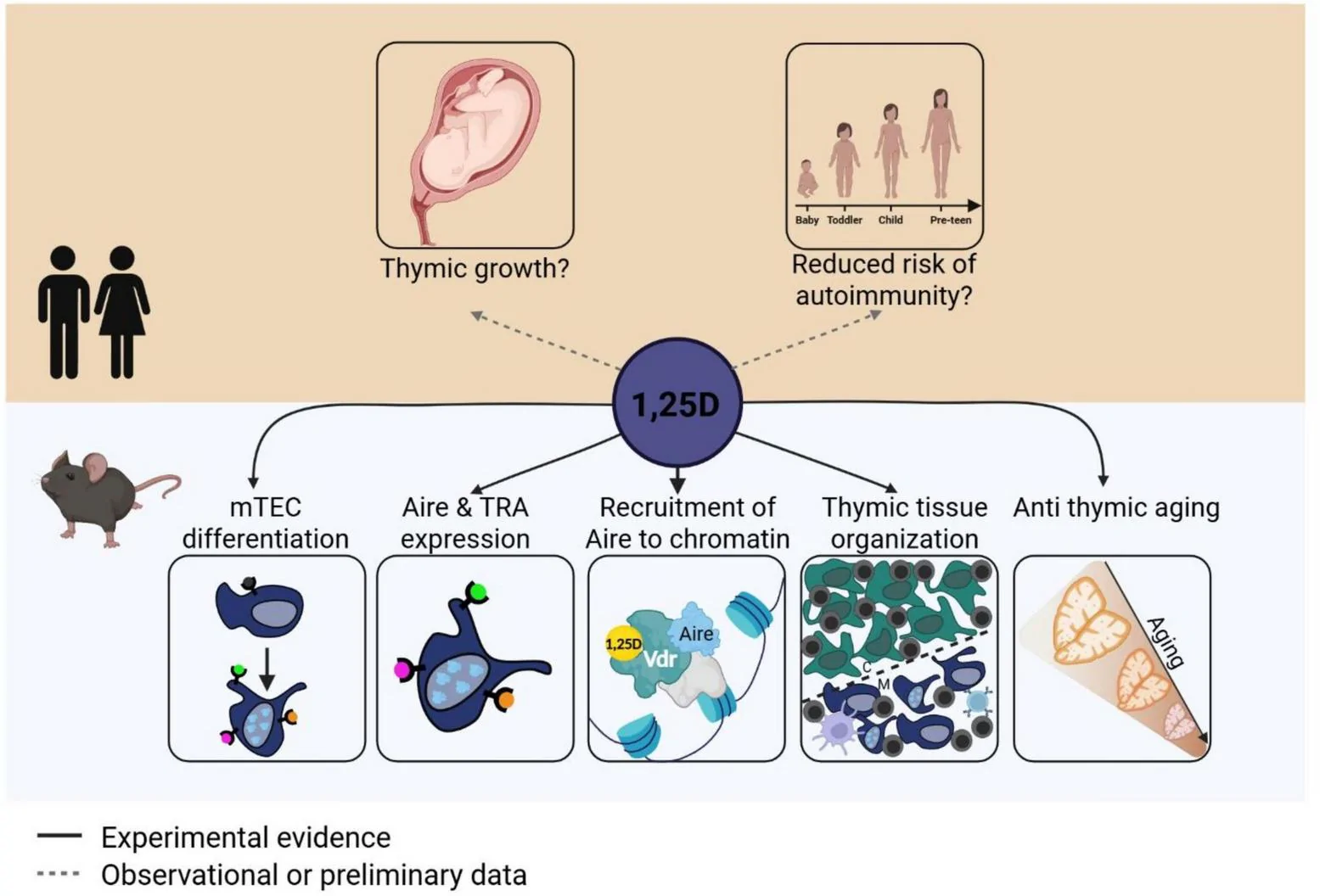
Overview of the effects of vitamin D on thymic biology. Associations between maternal vitamin D status and human fetal thymic size have been made. Observational studies found a link between vitamin D supplementation during infancy and future risk of developing type 1 diabetes. In mice, 1,25D regulates the differentiation of medullary thymic epithelial cells (mTECs) to Aire-expressing cells, as well as the expression of Aire and TRAs. Agonist-bound Vdr recruits Aire to chromatin where it can exert its transcriptional effects. Loss of 1,25D results in disorganized cortico-medullary boundaries and accelerates aging-associated thymic involution. See text for details.

## Aire and the Vdr

Despite the importance of Aire, it’s precise mechanism of action and targets remain incompletely understood. It contains a CARD domain, SAND domain, and two PHD finger domains (39). While SAND domains can interact with DNA, Aire does not appear to interact with DNA in a sequence-specific manner (40). This observation, in combination with data from transiently transfected cells showing that Aire target genes vary depending on the cell type it is expressed in Guerau-de-Arellano et al. (41), suggest that recruitment of Aire to DNA and its transcriptional activity are guided by its binding partners. In addition to its CARD and SAND domains, which confer protein binding ability, mouse and human AIRE also contain 4 LXXLL motifs (where x = any amino acid) which bind to agonist-bound nuclear receptors (42). Aire^+^ mTECs express mRNA for a number of nuclear receptors including the *Vdr* (34). In 2023, we found in transient expression experiments that the Vdr and Aire interact with each other in a 1,25D-dependent manner (34). In addition, chromatin immunoprecipitation assays revealed 1,25D-dependent recruitment of Aire to Vdr-target genes in mouse mTECs. Finally, Aire was found to be a coactivator of the Vdr. Together these data suggest that vitamin D signaling in mTECs drives an interaction between the Vdr and Aire resulting in its recruitment to chromatin and induction of gene expression (Figure 4). The implication here is that Vdr signaling may be one mechanism by which Aire is recruited to target genes to induce TRA expression.

## Premature thymic aging in vitamin D-deficient mice

As previously mentioned, the thymus is the most rapidly aging organ in the body, which has consequences for both anti-cancer immunity and pathogen clearance. While the thymi of wildtype and mutant 4-week-old mice were similar in size, surprisingly, we found that 8–10-week old *Vdr*- and *Cyp27b1*-deficient thymi were significantly smaller and had reduced cell counts when compared to wildtype littermates (36). Comparison of single cell sequencing data with publicly available datasets revealed striking similarities between differences in wildtype versus *Cyp27b1* KO transcriptional profiles and those of thymi of control versus lymphocytic choriomeningitis virus infection (LCMV)-infected mice and thymi of young versus old mice. Like thymic aging, LCMV infection induces thymic atrophy (43). Notably, TEC populations in aged thymic samples resembled those of *Cyp27b1* KO mice (36). In the stroma of *Cyp27b1*-deficient mice, we observed a blurring of cortico-medullary boundaries, a phenotype present in aged thymi.

Following up on this data, we observed that *Cyp27b1*-deficient thymi underwent accelerated thymic involution such that null thymi had 90% reduced cell counts at 26-weeks, relative to 4-week-old mice, whereas cell counts from wildtype littermate controls were only reduced by 18%. In addition, TECs from *Cyp27b1*-deficient mice had reduced expression of thymic longevity factors such as *Igf1*, *Fgf7*, and *Fgf21*, and reduced expression of proliferative markers including Ki67. These data indicate that besides the role of vitamin D in central tolerance, it plays an important role in delaying thymic involution through early adulthood (Figure 4).

## Vitamin D signaling in human thymic health and longevity: a knowledge gap

While human life expectancy has increased globally since the beginning of the millennium, the gap between life expectancy and healthy life span free of chronic disease has widened. By 2050, 22% of the world’s population is projected to be over 65, underlining the major public health challenge presented by an aging population (44). The pathophysiology of aging is characterized by an accumulation of damage and decline of function at the molecular and cellular levels (45). Cumulatively, this leads to increased rates of several diseases including diabetes, neurological and cardiovascular disorders and cancer, and is accompanied by physical and in some cases cognitive decline. Importantly, multiple pathologies associated with aging are linked to chronic inflammation, a condition that has been called inflammaging (46). In surveying the mechanisms of aging, Lopez-Otin et al. proposed that each hallmark of aging presented an opportunity to “decelerate, halt, or reverse aging by therapeutic interventions on the hallmark” (45). Thus, targeting the immune system to disrupt chronic inflammation associated with aging has considerable potential.

Within the aging immune system there is growing evidence for a skewing of hematopoietic stem cell differentiation in favor of cells of the myeloid lineage (47, 48). In addition, B cell production declines with age and there is a rise in the numbers of anergic B cells. The increase in the myeloid to lymphoid cell ratio is further exacerbated by a decline in T cell production arising from age-related thymic involution (29, 44). Robust T cell responses are critical for protection against infections and cancer progression. In addition, T cells can recognize and eliminate senescent cells (49), whose production rises with aging (45). Thus, reversal of the myeloid-lymphoid cell imbalance associated with aging, with an emphasis on maintaining or enhancing T cell production, may be key to reducing inflammation and protecting against the development of age-related diseases.

As discussed above, the thymus is essential for establishment of a highly diverse repertoire of TCRs generated during a multi-step maturation process. Although the size of the thymus in humans peaks with adolescence, accumulating evidence supports the importance of thymic longevity in maximizing the healthy life span. With progressive thymic involution in both humans and mice, production of so-called recent thymic emigrant T cells declines and along with it a reduction in the number naïve T cells (50–52), leading to a narrowing of the T cell repertoire (53, 54). This decline in TCR diversity and loss of naïve T cells can lead to a weakened response to previously encountered antigens (55, 56). Collectively, this leads to diminished protection against pathogens and attenuated vaccine responses, along with increased risk of tumour development and autoimmunity (57).

In young adult mice, thymectomy accelerates the accumulation of senescence-associated T cells, whereas embryonic thymic implantation in older adult mice attenuates the process (58). Consistent with this, children who have undergone thymic resection during cardiac surgery subsequently display a peripheral T-cell repertoire more similar to that of 75-year-olds than young adults (59). A large-scale study of the health consequences of thymectomy in adults revealed an increase in all-cause mortality in thymectomized patients relative to a control population 5 years after surgery. Similarly, thymectomy led to an increased risk of cancer, as well as elevated levels of autoimmunity in a subgroup of patients after elimination of individuals with preoperative infection, cancer or autoimmunity (60). These findings are bolstered by recent studies using thymic health as a measure of healthy aging. Thymic health was assessed using a deep-learning AI algorithm to analyze CT (computed tomography) images from over 5,000 individuals. The algorithm generated a “health score” derived from thymic size and was used to analyze large groups from two clinical trials. Notably, thymic health was better in women than men, and higher scores were associated with reduced risk of development of lung cancer, heart, digestive and lung diseases as well as metabolic disorders such a diabetes (61). The group also showed that thymic health score is strongly associated with efficacy of immunotherapy for several cancer types (62, 63). In a lung cancer study examined in the same paper, health score correlated with T cell receptor diversity (62).

The above, coupled with our results revealing that loss of vitamin D signaling leads to premature thymic involution in mice (36), suggest that it would be of great interest to understand if (changes in) vitamin D status during adulthood would affect thymic “health score” and longevity in humans. In this regard, while clinical studies of the effects of vitamin D supplementation on mortality have provided mixed results (64, 65), likely because of supplementation of already sufficient populations, others have found a link between vitamin D deficiency and increased risk of all-cause mortality (66–69). Unfortunately, while relatively little was known about vitamin D signaling thymus prior to our studies in the mouse (34, 36), there is no published data that we are aware of on the molecular mechanisms of vitamin D action in human thymus. We found VDR transcripts in publicly available databases from single cell RNA sequencing derived from thymi of two 10-month-old infants (34). We also identified *CYP27B1-* and *CYP24A1-*positive TEC populations in the same databases, consistent with the formation of 1,25D and VDR-regulated transactivation in human TECs. In addition, the VDR and CYP27B1 are present in human infant TEC populations as detected by immunofluorescence microscopy (Artusa and White, unpublished). These preliminary data indicate the presence of vitamin D signaling machinery, as well as the means to produce 1,25D, in human pediatric TECs. It would be of interest to further characterize protein level expression of the VDR and key metabolic enzymes prenatally, as well as throughout childhood and adulthood. Moreover, rigorous assessment of the presence of active vitamin D signaling in *in vitro* experiments, and whether this has a role in AIRE expression or mTEC development in humans is warranted.

There is some published evidence that vitamin D supplementation can affect thymic health early in life. Two studies provided links between decreased maternal serum 25D concentrations and diminished fetal thymic perimeter (70, 71). In a study of 44 infants (22 malnourished, 22 healthy; 2–6 months), malnutrition was associated with a reduced thymic index (organ length × breadth). In the malnourished cohort, vitamin D supplementation over 8 weeks led to a statistically significant increase in thymic index (72). Collectively, these data suggest that vitamin D signaling is active in the human thymus, at least in infants, and that vitamin D status may affect thymic health in this population. However, unlike in the mouse, no mechanistic basis for any of these observations in human samples has been provided thus far. Finally, there is no data we are aware of addressing links between vitamin D status and thymic health/longevity in adult human populations. This, in our view, given the clinical links between vitamin D deficiency and mortality, represents a substantial knowledge gap that should be addressed in clinical studies.

## Data Availability

The original contributions presented in this study are included in this article/Supplementary material, further inquiries can be directed to the corresponding author.
